# Antiviral activity of micafungin against enterovirus 71

**DOI:** 10.1186/s12985-016-0557-8

**Published:** 2016-06-13

**Authors:** Chonsaeng Kim, Hyunju Kang, Dong-eun Kim, Jae-Hyoung Song, Miri Choi, Mingu Kang, Kyungjin Lee, Hae Soo Kim, Jin Soo Shin, Hyejeong Jeong, Sunhee Jung, Sang-Bae Han, Jong Heon Kim, Hyun-Jeong Ko, Chong-Kyo Lee, Meehyein Kim, Sungchan Cho

**Affiliations:** Anticancer Agent Research Center, Korea Research Institute of Bioscience & Biotechnology, 30 Yeongudanji-ro, Ochang-eup, Cheongwon-gu, Cheongju-si, Chungcheongbuk-do 28116 South Korea; Department of Biomolecular Science, Korea University of Science and Technology, 217 Gajeong-ro, Daejeon, 34113 South Korea; Virus Research and Testing Center, Korea Research Institute of Chemical Technology, 141 Gajeong-ro, Yuseong-gu, Daejeon, 34114 South Korea; Laboratory of Microbiology and Immunology, College of Pharmacy, Kangwon National University, 1 Gangwondaehak-gil, Chuncheon-si, Gangwon-do 24341 South Korea; College of Pharmacy, Chungbuk National University, 1 Chungdae-ro Seowon-gu, Cheongju-si, Chungcheongbuk-do 28644 South Korea; Cancer Cell and Molecular Biology Branch, Research Institute, National Cancer Center, 323 Ilsan-ro, Ilsandong-gu, Goyang-si, Gyeonggi-do 10408 South Korea

**Keywords:** Enterovirus, Enterovirus 71 (EV71), Micafungin, FDA-approved drug, Antiviral drug

## Abstract

**Background:**

Enterovirus 71 (EV71) is a major causative agent of hand-foot-mouth disease (HFMD) and also causes severe neurological complications, leading to fatality in young children. However, no effective therapy is currently available for the treatment of this infection.

**Methods:**

We identified small-molecule inhibitors of EV71 from a screen of 968 Food and Drug Administration (FDA)-approved drugs, with which clinical application for EV71-associated diseases would be more feasible, using EV71 subgenomic replicon system. Primary hits were extensively evaluated for their antiviral activities in EV71-infected cells.

**Results:**

We identified micafungin, an echinocandin antifungal drug, as a novel inhibitor of EV71. Micafungin potently inhibits the proliferation of EV71 as well as the replication of EV71 replicon in cells with a low micromolar IC_50_ (~5 μM). The strong antiviral effect of micafungin on EV71 replicon and the result from time-of-addition experiment demonstrated a targeting of micafungin on virion-independent intracellular process(es) during EV71 infection. Moreover, an extensive analysis excluded the involvement of 2C and 3A proteins, IRES-dependent translation, and also that of polyprotein processing in the antiviral effect of micafungin.

**Conclusions:**

Our research revealed a new indication of micafungin as an effective inhibitor of EV71, which is the first case reporting antiviral activity of micafungin, an antifungal drug.

**Electronic supplementary material:**

The online version of this article (doi:10.1186/s12985-016-0557-8) contains supplementary material, which is available to authorized users.

## Background

Enterovirus 71 (EV71) is one of the major etiological agent of hand-foot-mouth disease and also causes severe neurological symptoms, such as aseptic meningitis, encephalitis and acute flaccid paralysis, which can lead to even death [1–4]. Since its first discovery in 1969, EV71 outbreak has occurred frequently in the Asia-Pacific region and caused hundreds of annual deaths. Despite its enormous threat to public health, currently no effective vaccines or therapeutic drugs are yet available.

EV71 is a member of human enterovirus A (HEV-A) species under genus *Enterovirus* in the *Picornaviridae* family [5]. EV71 is a small and non-enveloped virus with a positive-sense single-stranded RNA genome of 7500–8000 nucleotides that is composed of a long open reading frame (ORF) flanked by 5’ and 3’nontranslated regions (NTR) [2, 6]. First, virus particle attaches and enters into host cells via specific receptors, and then the viral RNA genome is released into the cytoplasm. The viral RNA is used as mRNA for the initiation of translation at the internal ribosomal entry site (IRES) in the 5’ NTR, producing a large polyprotein. The viral polyprotein is further cleaved into individual viral proteins (VP4, VP2, VP3, VP1, 2A^pro^, 2B, 3A, 3B, 3C^pro^, and 3D^pol^) by two viral proteases 2A^pro^ and 3C^pro^. Negative-sense RNA genomes are also generated mainly by the action of 3D^pol^ and serve as templates for the production of positive-sense RNA genomes [6]. Amplified positive-sense RNA genomes are packaged by structural proteins (VP1, VP2, VP3, and VP4) to produce infectious viral particles and then released from the host cell.

Effective antiviral drugs for the treatment of various diseases associated with enteroviral infection have been enthusiastically explored. Currently, many synthetic compounds (Gemcitabine [7], Pleconaril [8, 9], CsA [10], BPROZ [11], GPP3-1 [12], LVLQTM [13], Enviroxime [14], rupintrivir [15], DTrip-22 [16], and aurintricarboxylic acid [17]) and natural products (lycorine [3], raoulic acid [18], chrysin [19], and ginsenosides [20, 21]) have been reported to have inhibitory activities against part of or broad range of enteroviruses. However, none of them has been demonstrated to be sufficiently effective at the clinical level. Undesirable side effects in vivo are another limiting factor for the therapeutic application of those compounds. Therefore, the development of new anti-enteroviral drug candidates are urgently required before the enteroviruses cause more severe health problems in human society. In this regard, we chose FDA-approved drugs with proven clinical safety, with which new clinical application for EV71-associated diseases would be more favorable, for screen of anti-EV71 chemicals.

Here, we identified micafungin as an effective inhibitor of EV71 from a screen of 968 FDA-approved drugs. Micafungin potently inhibited the proliferation of EV71 in LLC-MK2 Derivative cells and moderately inhibited that of Coxsackievirus B3 (CVB3) in HeLa cells. Moreover, micafungin showed a strong inhibitory effect on the replication of EV71 replicon in Vero cells, indicating its effect on intracellular process(es) that are independent of the virus particle. As for the mode of action, further analysis ruled out the involvement of some of intracellular process(es), such as polyprotein processing by 3C^pro^, IRES-dependent translation, and 2C and 3A proteins, in the antiviral action of micafungin.

## Methods

### Cells, viruses, and chemicals

Vero, HeLa, 293 T, LLC-MK2 Derivative, and H1HeLa cells were used as described previously [7]. EV71 (strain BrCr) (ATCC VR-1775), EV71 (strain H) (ATCC VR-1432) were purchased from ATCC, and EV71 (strain 1095) was kindly provided by Yorihiro Nishimura [22]. Those were expanded in LLC-MK2 Derivative cells. CVB3 and Human rhinoviruses were used as previously described [7]. FDA-approved drug library version 2 was purchased from Enzo life science for screen of antiviral compound. Micafungin (Selleckchem), and Rupintrivir (Santa Cruz) were purchased and dissolved in DMSO for further analysis.

### Antibodies

Anti-flag and -β-actin monoclonal antibodies were obtained from Sigma-Aldrich. EV71 3C antibody (GTX630191) was purchased from GeneTex. Anti-dsRNA J2 mouse monoclonal antibody was purchased from English & Scientific Consulting Kft. Secondary antibodies conjugated to horseradish peroxidase or Alexa Fluor 488 were purchased from Thermo Fisher Scientific and Life Technologies, respectively.

### Replicon assay

Plasmids pRibFluc-EV71 wt and p53CB3-LUC, which contains the firefly luciferase gene in place of the P1 capsid coding region of EV71 and CVB3 viral genome, were generously provided by Frank J. M. van Kuppeveld (Utrecht University, Netherlands). Plasmids pRib-LUC**-**CB3/T7-wt [23–25] and pRib**-**LUC**-**CB3*/*T7-(2C-A224V, I227V, A229V or 3A-H57Y) mutant clones [25, 26], were also used. Ribomax large-scale RNA production system (Promega) was used for the synthesis of replicon RNAs [7]. To perform a screen of 968 FDA-approved drugs (Selleckchem), Vero cells (3 × 10^5^ cells/well) were transfected with 0.4 μg of the EV71 or CVB3 replicon RNAs using Lipofectamine 2000 (Invitrogen), split into 96 well plates (2 × 10^4^ cells/well), and simultaneously treated with 10 μM of chemicals in 1 % DMSO solution. Eight hours after treatment of EV71 and CVB3 replicons, cells were assayed for firefly luciferase activity using One-Glo Luciferase Assay System (Promega). CellTiter-Glo Luminescent Cell Viability Assays (Promega) was used for measuring cytotoxicity.

### Antiviral activity assay

Antiviral activity of chemicals was tested as previously described [7]. Mock-infected and DMSO (1 %)-treated cells were regarded as a 100 % survival and virus-infected and DMSO (1 %)-treated cells were regarded as a 0 % survival. The antiviral activity of compounds was calculated as the percentage of the control.

### Cell toxicity assay

MTT assay or CellTiter-Glo Luminescent Cell Viability Assay Kit (Promega) was used for measuring cell toxicity as described previously [7]. Cell toxicity was calculated as the percentage of the control.

### Immunofluorescence microscopy

Cells in 96-well plate were infected with EV71 (1 MOI) and treated with micafungin. At 20 h after infection, cells were fixed and permeabilized with a 3:1 mixture of ice-cold methanol-acetone. Infected cells were stained with anti-dsRNA antibody and anti-mouse secondary antibody conjugated with Alexa Fluor 488 and followed by counterstaining with 4′,6-diamidino-2-phenylindole (DAPI) (Product # 62248, Thermo Scientific). Operetta system (Perkin Elmer) was used for capturing images. Viral infection was quantified using HARMONY software in Operetta system. The ratio of infection was calculated as the percentage of the control.

### Western blotting and RT-PCR

Virus-infected samples were prepared as described in immunofluorescence microscopy section. Western blotting was performed as previously described [27]. Twenty hours after infection, total cell lysates were harvested and analyzed using anti-3C and -β-actin antibodies. RT-PCR was performed as previously described [28]. For RT-PCR of viral RNA, total cellular RNAs were purified using QIAGEN RNeasy Mini Kit according to manufacturer’s manual. Reverse transcription was performed with random hexamer and SuperScript III reverse transcriptase (Invitrogen). The region spanning from 3B to 3C of EV71 was amplified with primer csp120 (5’-TTGAACCTTAGTGGTAAGCCCAC-3’) and csp121 (5’-GTGATTGATCCCTTCTATGAG-3’) and Accupower PCR premix (Bioneer). The β-actin mRNAs were also analyzed as a loading control.

### Time-of-addition assay

Micafungin (20 μM) and rupintrivir (4 μM) were added to LLC-MK2 Derivative cells at indicated time points before or after EV71 infection. At 20 h post-infection, cells were fixed and stained with anti-dsRNA antibody and AF488-conjugated anti-mouse secondary antibody. Nuclei were counterstained with DAPI. The quantification of viral infection was done as described in immunofluorescence microscopy section. Viral infection was calculated as the percentage of the control.

### IRES assay

Dual luciferase reporter plasmids pR/EV71(strain BrCr)/F-PEST and pR/CVB3/F-PEST were exploited and IRES assay was performed as previously described [7].

### 3C protease assay

293 T cells were transfected with pcDNA3-flag-3CD [7] using X-tremeGENE DNA Transfection Reagent (Roche), maintained for 15 h, and treated with 10 μM of micafungin for another 9 h before being subjected to Western blotting with anti-flag antibody.

## Results

### Screening of anti-EV71 compounds from FDA-approved drugs

**Fig. 1:**
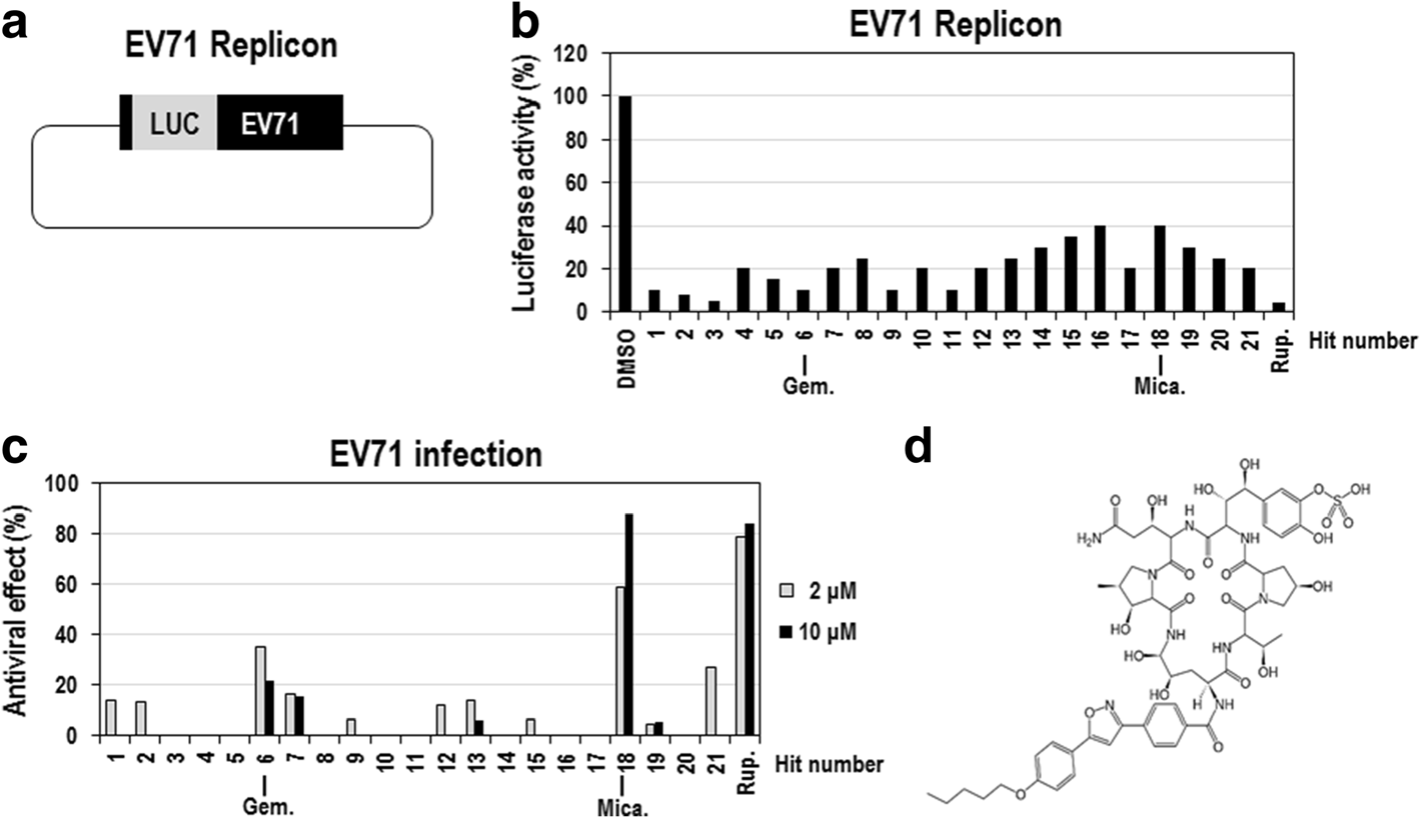
Identification of micafungin as an anti-EV71 inhibitor from a screen of the FDA-approved drug library. **a** Schematic diagram of DNA encoding the EV71 replicon. **b** Vero cells were transfected with in vitro-transcribed EV71-replicon RNAs, immediately treated with 968 FDA-approved drugs (10 μM) for 8 h, and then assayed for firefly luciferase activity. Rupintrivir (10 μM) was used as a positive control. The luciferase activities from cells treated with 21 primary hits including micafungin were presented in graph. The luciferase activity from DMSO-treated cells was considered to be 100 %. **c** The antiviral activities of the primary hits were further evaluated in EV71-infected LLC-MK2 Derivative cells. The LLC-MK2 Derivative cells were infected with EV71 (100 CCID_50_), simultaneously treated with the 21 primary hits (2 and 10 μM) for 96 h, and then cell viabilities were analyzed by using MTT assay. Rupintrivir (2 and 10 μM) was used as a positive control. The viability of DMSO-treated cells was considered to be 0 %, and that of uninfected cells was considered to be 100 %. **d** The chemical structure of micafungin

Many compounds have been reported to have anti-EV71 activity in vitro, but their clinical potential needs to be extensively evaluated. To date no compound has been proved to be safe and effective in clinical setting. Therefore, we sought to identify new drug candidate(s) from FDA-approved drugs, with which clinical application for EV71-associated diseases would be more favorable, using EV71 subgenomic replicon system. This replicon system contains a firefly luciferase gene in place of the P1 structural genes (VP4-VP1), which allows easy and quantitative measurements of intracellular viral replication (Fig. 1a) [23, 24]. According to the preliminary experiments, luciferase activity from Vero cells transfected with in vitro-transcribed EV71-replicon RNAs increased in a time-dependent manner and reached a maximum at 10 h after transfection (Additional file 1: Figure S1). Therefore, we chose to screen the compounds for antiviral activity by applying them to the cells for 8 h. Primary screening of 968 FDA-approved drugs (10 μM) in Vero cells transfected with EV71 replicon RNAs identified 21 compounds that significantly decreased the luciferase activity (more than 60 % reduction compared with DMSO-treated control; Fig. 1b).

Next, to examine whether primary hits inhibit the proliferation of intact EV71 in cells, EV71 (BrCr)-infected LLC-MK2 Derivative cells were treated with 2 and 10 μM concentrations of the 21 identified compounds for 96 h and then analyzed by MTT assay, which is prevalently used for quantitative measurement of cell viability. In this assay, EV71 infection causes a cytopathic effect (CPE) on the LLC-MK2 Derivative cells, reducing the cell viability by almost 100 %, and any compound with antiviral activity can cause a relative increase in the cell viability. Among 21 primary hits tested, the treatment with micafungin most highly increased the cell viability by more than 60 % compared with the DMSO-treated control (Fig. 1c). In the same condition, rupintrivir, a potent 3C inhibitor, showed strong antiviral activity as previously reported [15]. Gemcitabine, recently identified as an anti-enteroviral compound [7], also showed a moderate anti-EV71 activity. Intriguingly, micafungin is a well-known antifungal drug and has never been reported to have any antiviral activity. Thus, we decided to further investigate its antiviral activity (Fig. 1d).

### Micafungin potently inhibits the proliferation of EV71 in mammalian cells

**Fig. 2:**
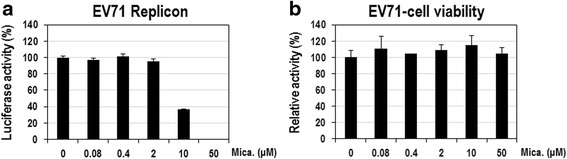
Micafungin potently inhibits the replication of the EV71 replicon. **a** Vero cells were transfected with in vitro-transcribed EV71-replicon RNAs, simultaneously treated with the indicated concentrations of micafungin for 8 h, and then assayed for firefly luciferase activity. The luciferase activity of DMSO-treated cells was considered to be 100 %. **b** In the same condition, another set of EV71 replicon-transfected cells was assayed for cell viability using CellTiter-Glo reagent. The activity of DMSO-treated cells was considered to be 100 %

To examine how strongly micafungin inhibits the replication of EV71 in cells, Vero cells were transfected with in vitro*-*transcribed EV71-replicon RNAs, immediately treated with a various concentrations of micafungin for 8 h, and then assayed for luciferase activity. Micafungin had IC_50_ of ~5-8 μM for replication of EV71 replicons (Fig. 2a). In the same condition, it had little cytotoxic effect in Vero cells, which was assessed using CellTiter-Glo reagent (Fig. 2b). Similar inhibitory effects were also observed in Vero cells transfected with CVB3-replicon RNAs (Additional file 2: Figure S2A). Low cytotoxicity of micafungin in Vero cells was also confirmed in an experiment with a longer treatment lasting 24 h (Additional file 3: Figure S3A) and 48 h (Additional file 3: Figure S3B).

**Fig. 3:**
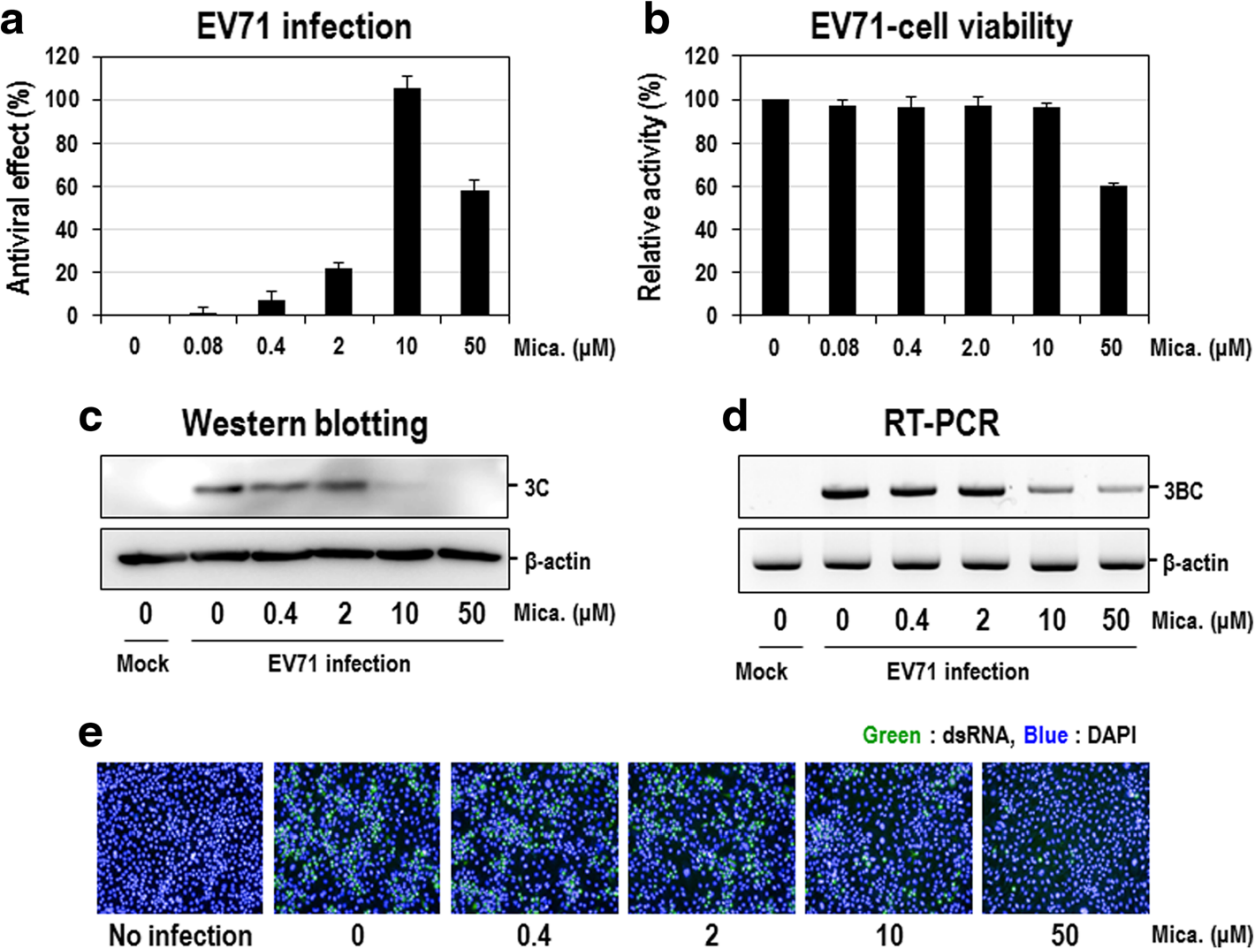
Micafungin potently inhibits EV71 proliferation in LLC-MK2 Derivative cells. **a** LLC-MK2 Derivative cells were infected with EV71 (100 CCID_50_) and immediately treated with increasing concentrations of micafungin. Four days after treatment, antiviral activity was determined by the reduction of the cytopathic effect in an MTT assay. Cell viability of DMSO-treated cells was set to 0 % and that of uninfected cells was set 100 %. **b** Same cells treated with indicated concentrations of micafungin without EV71 infection were also analyzed for cell viability by using MTT assay. **c-e** LLC-MK2 Derivative cells were infected with EV71 (1 MOI) and simultaneously treated with increasing concentrations of micafungin. **c** Twenty hours post-infection, total cell extracts were prepared from cells and subjected to Western blot analysis with anti-3C antibody. β-actin was also analyzed as a loading control. **d** Total RNAs were prepared from cells in (**c**) and then subjected to RT-PCR for 3BC region of EV71 viral RNA. β-actin mRNAs were also analyzed as a negative control. **e** Twenty hours post-infection, dsRNAs were stained by using specific antibody and visualized by FITC-conjugated secondary antibody (green). Nuclear DNA was also visualized by DAPI staining (blue)

The antiviral effect of micafungin was further analyzed in EV71-infected LLC-MK2 Derivative cells. Cells were infected with EV71 (strain BrCr) (hereafter referred to as EV71), simultaneously treated with a broad range of concentrations of micafungin for 96 h, and then analyzed by MTT assay. Micafungin exhibited an obvious dose-dependent antiviral activity, with an estimated EC_50_ of ~5 μM (Fig. 3a), which is quite similar to that obtained from replicon assay (Fig. 2a). Apart from this obvious antiviral activity, there was a slight decrease in cell viability (Fig. 3b) and even in antiviral activity (Fig. 3a) at 50 μM, indicating the possible cytotoxicity. Given that micafungin had little cytotoxic effect in Vero cells treated for 24 or 48 h (Additional file 3: Figure S3), micafungin might have a mild cytotoxicity, particularly at a higher concentration and longer treatment condition. Similar antiviral activity was also shown in LLC-MK2 Derivative cells infected with EV71 (strain H isolated from Chinese patient) or EV71 (strain 1095) genotype C2, even though maximal efficacy was lower than that for EV71 (strain BrCr) (Additional file 4: Figure S4).

To further confirm the antiviral activity of micafungin in EV71-infected cells, viral proteins and RNAs were analyzed. EV71-infected LLC-MK2 Derivative cells were treated with a broad range of concentrations of micafungin for 20 h, and then the total cell extracts and RNAs were prepared and analyzed by Western blot with anti-3C antibody and RT-PCR with primers corresponding to the 3B-3C regions, respectively. The amount of EV71 3C protein decreased by the treatment with micafungin in a dose-dependent manner and is hardly detected at 10 μM (Fig. 3c), which is similar to those observed in EV71-infected cells and replicon assays (EC_50_ = 2–10 μM). Similar antiviral effects were also shown in the viral RNA analysis. The amount of EV71 RNAs were dramatically reduced by the micafungin treatment with an estimated EC_50_ of ~5 μM (Fig. 3d).

Moreover, the strong antiviral activity of micafungin was further confirmed by another approach, in which EV71-infected LLC-MK2 Derivative cells were visualized by staining the dsRNA with an antibody conjugated with a fluorescent dye, and quantified by counting the stained cells. EV71-infected cells exhibited a strong fluorescent signal of the dsRNA and then gradually decreased by a dose-dependent treatment of micafungin, with an estimated IC_50_ of 10 μM (Fig. 3e and Additional file 5: Figure S5). Collectively, these results clearly demonstrated that micafungin is a strong inhibitor of EV71.

### Micafungin has a moderate antiviral activity against other enteroviruses

In order to examine whether micafungin has an antiviral effect on a broad spectrum of enteroviruses, we tested other enteroviruses such as Coxsackievirus group B type 3 (CVB3) and human rhinovirus (HRV), which are also single-strand, positive-sense enteroviruses that are significantly affecting public health. CVB3, one of the most well-studied enteroviruses and a member of HEV-B, is one of the main causes of viral meningitis, myocarditis, and pancreatitis [29, 30]. HRV, the predominant cause of the common cold, also belongs to the genus *Enterovirus* along with EV71 and CVB3 [7]. Micafungin had an antiviral effect in CVB3-infected HeLa cells, but its activity was much weaker than that observed in EV71-infected LLC-MK2 Derivative cells (compare Fig. 3a and Additional file 6: Figure S6A). Similarly, weak antiviral activities of micafungin were also shown in H1HeLa cells infected with three different types of HRV (HRV-14, HRV-21, and HRV-71) (Additional file 7: Figure S7).

### Micafungin affects virion-independent intracellular processes during EV71 infection

**Fig. 4:**
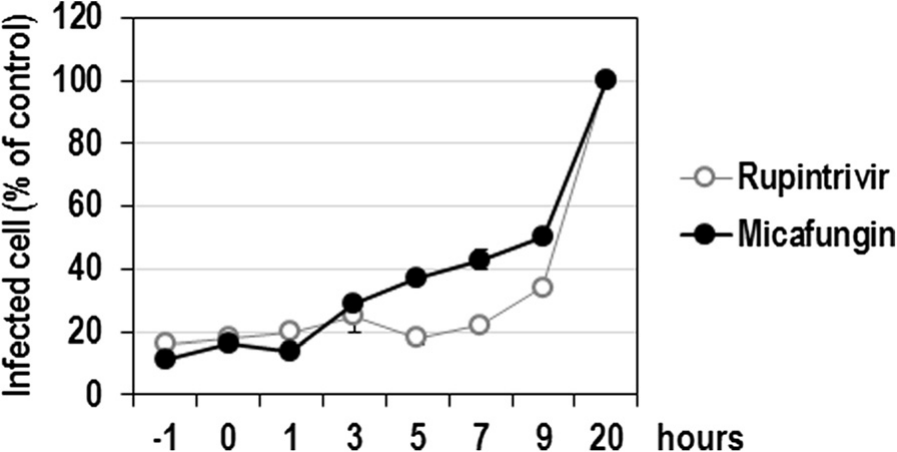
Antiviral activity of micafungin depending on the time of addition. LLC-MK2 Derivative cells were infected with EV71 at 1 MOI and treated with 20 μM of micafungin or 4 μM rupintrivir at the indicated times prior to or after virus infection. Twenty hours post-infection, virus-infected cells were visualized by staining with anti-dsRNA antibody and percentage of infected cells out of total cells were calculated

The inhibitory effect of micafungin might affect any step in the infectious cycle, including attachment, entry, uncoating, translation, polyprotein processing, replication, assembly, and release. According to the results in Fig. 2, the inhibitory effect of the replication of the EV71 replicon by micafungin provides a clear evidence that micafungin targets intracellular step(s), which are independent of the virus particle, such as translation, polyprotein processing, and replication. To further confirm this observation, micafungin was applied to the culture medium at various time points during virus infection (−1, 0, 1, 3, 5, 7, 9 and 20 h), and the antiviral effect of micafungin was assessed by quantifying the infected cells showing a fluorescent signal of viral dsRNA. The strong antiviral effect of micafungin was also shown when the drug was added at 1 h post-infection, and that effect was nearly as strong as that obtained when the drug was added prior to or at the time of infection (−1 or 0 h; Fig. 4). Considerable antiviral effect of micafungin sustained until 9 h post-infection. Overall, time-course effect of micafungin was quite similar to that of rupintrivir, a well-known 3C inhibitor. Consistent with the observation from replicon assay, these results indicate that micafungin inhibits the proliferation of EV71 most possibly by targeting any step(s) after entry in an early viral infection.

**Fig. 5:**
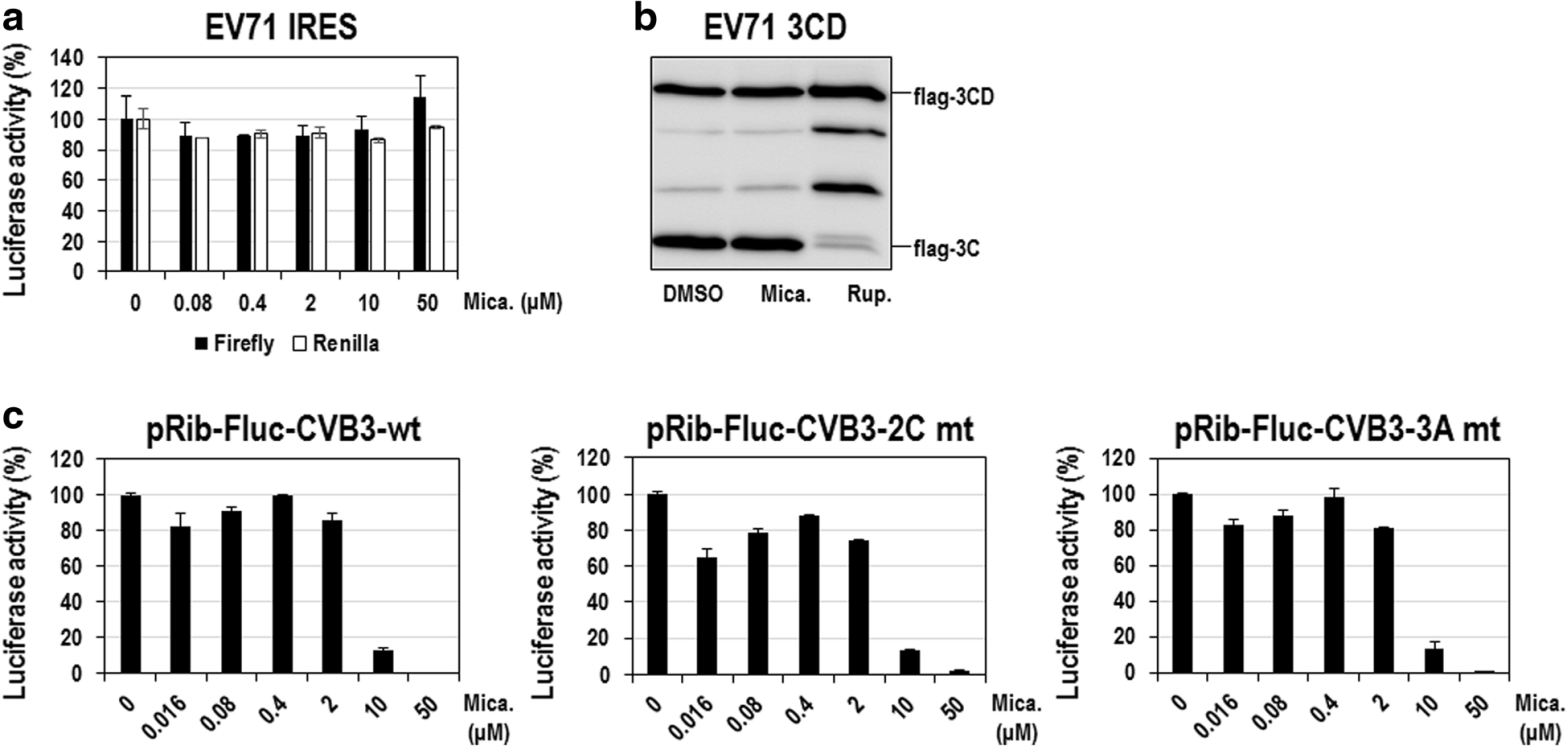
Anti-EV71 effect of micafungin is not related with IRES-dependent translation, polyprotein processing, and 2C and 3A. **a** 293 T cells were transfected with dual luciferase reporter DNA measuring EV71 IRES-dependent translation and then treated with indicated concentrations of micafungin. Twenty-four hours after compound treatment cells were assayed for firefly and *renilla* luciferases. Luciferase activities from DMSO-treated cells were set to 100 %. **b** 293 T cells were transfected with plasmid expressing flag-EV71(3CD) and then treated with 10 μM of micafungin. Nine hours after compound treatment total cell extracts were prepared and subjected to Western blot analysis with anti-flag antibody. Rupintrivir (10 μM) was included as a positive control. **c** Vero cells were transfected with in vitro transcribed CVB3-wt, CVB3-2C mt, or CVB3-3A mt replicon RNAs and simultaneously treated with indicated concentrations of micafungin. Eight hours after compound treatment cells were assayed for luciferase activity. Luciferase activities from DMSO-treated cells were set to 100 % for each replicons

In order to further define which step of EV71 infection, particularly among virion-independent intracellular processes, is targeted by micafungin, we developed extensive assays, as reported in our previous study [7], and examined the involvement of each process in EV71 inhibition by micafungin. First, the possibility that micafungin affects translation initiation directed by the IRES in the 5’ NTR of the EV71 RNA was examined. For this we used a dual-luciferase reporter system, in which the expression of the firefly and *renilla* luciferases is controlled by EV71 IRES-dependent and cap-dependent translation, respectively. As a result, micafungin had little effect on firefly luciferase activity in 293 T cells, excluding the involvement of EV71 IRES-dependent translation in the antiviral effect of micafungin (Fig. 5a). Next, to know whether an EV71 protease, 3C^pro^, can be targeted by micafungin, we examined the cleavage processing of 3CD. The cleavage pattern of 3CD precursor protein exogenously overexpressed in 293 T cells was not altered at all by the micafungin treatment (Fig. 5b), excluding the association of 3C protease with the inhibitory effect of micafungin. In contrast, treatment with rupintrivir, a 3C^pro^ inhibitor, obviously increased the amount of 3CD precursor protein with a reciprocal decrease of 3C protein level (Fig. 5b). In addition, the involvement of 2C and 3A proteins in the inhibitory effect of micafungin was also ruled out, because the replication of EV71 replicons containing well-characterized 2C or 3A drug-resistant mutations were also affected by micafungin to an extent similar to that, to which the replication of wild-type CVB3 replicon was affected (Fig. 5c). It should be noted that we took the advantage of 2C or 3A mutant CVB3 replicons, which were available, for the evaluation of micafungin because many inhibitors targeting 2C or 3A of CVB3 also have a similar effect on those of EV71 [26, 31]. Collectively, our mechanistic analyses showed that the antiviral effect of micafungin is not associated with IRES-dependent translation, polyprotein processing involving 3C^pro^, and 2C and 3A proteins.

## Discussion and conclusion

Even though there has been an increasing demand for antiviral therapy to control EV71 infections, no effective antiviral drugs of EV71 are currently available. Most of anti-EV71 inhibitors are only at the preliminary stages of drug development [6]. Moreover, even rapidly moving drug candidates such as rupintrivir (a potent 3C^pro^ inhibitor) and pleconaril (a coat protein binder) have been dropped from further clinical development because of concerns about viral resistance, side effects, or poor efficacy in patients [32]. Thus, the development of new antiviral drugs against EV71 is urgently required before EV71 causes more severe health problems in human beings. To efficiently achieve this goal, we searched for new antiviral compounds against EV71 from the FDA-approved drugs, which would facilitate the clinical application of drug candidate for EV71-associated diseases. In the present study, we identified micafungin, a drug for antifungal therapy, as an anti-EV71 agent. Extensive analysis in various assay systems explicitly demonstrated that micafungin is a potent inhibitor of EV71, which is the first report about its antiviral activity.

Micafungin is an FDA-approved antifungal drug for the therapy of diseases associated with Candida infection mostly. It is a large lipopeptide molecules with a complex aromatic side chain [33], and is classified into echinocandin family which inhibits the synthesis of the cell wall by targeting β-1,3-D-glucan synthase of fungi [34]. Even though fungi are eukaryotes like human beings, the cell wall is not shared by either mammalian cells or viruses [35]. In this regard antiviral effect of micafungin does not seem to be related with inhibition of β-1,3-D-glucan synthase. Unfortunately, more biological activities of micafungin have not been defined yet, thus it is not easy to explain the precise mechanism of antiviral effect on EV71. Nevertheless, based on our extensive analysis we could speculate the possible mode of action that micafungin probably targets intracellular event during EV71 infection such as translation, polyprotein processing, replication, or other such processes rather than virion-dependent processes such as viral entry and assembly. In the following studies, the underlying mechanism of antiviral effect by micafungin will be further investigated through the identification of viral mutation(s) conferring viral resistance to the treatment with micafungin. Moreover, it will be worthy to test the possible antiviral activities of caspofungin and anidulafungin, which are also antifungal drugs in echinocandin family [33, 35–37].

In this study, we provide a new function of micafungin, the drug currently being used for antifungal therapy, as an effective antiviral inhibitor of life-threatening EV71. Further validation in virus-infected animal models remains to suggest micafungin as an anti-EV71 drug candidate. Given that micafungin has been recently reported to show some hepatotoxic side-effects in vivo, the effectiveness at a low dose will be required.

## Additional files

Additional file 1: Figure S1. Time-dependent luciferase activity of EV71 replicon in Vero cells. Vero cells were transfected with in vitro transcribed EV71-replicon RNAs and then firefly luciferase activity was measured at an interval of two hours. (TIF 80 kb) Additional file 2: Figure S2. Micafungin inhibits the replication of CVB replicon. (A) Vero cells were transfected with in vitro transcribed CVB3 replicon RNAs, instantly treated with indicated concentrations of micafungin for 8 hours and then assayed for firefly luciferase activity. Luciferase activity of DMSO-treated cells was set to 100 %. (B) At the same condition another set of CVB3 replicon-transfected cells were assayed for cell viability by using CellTiter-Glo reagent. Activity of DMSO-treated cells was set to 100 %. (TIF 100 kb) Additional file 3: Figure S3. Analysis of the cell toxicity of micafungin. Vero cells were treated with the indicated concentrations of micafungin for 24 h or 48 h and then assayed for viability using CellTiter-Glo reagent. The activity of DMSO-treated cells was considered to be 100 %. (TIF 98 kb) Additional file 4: Figure S4. Antiviral effect of micafingin on EV71 (strains H and 1095). LLC-MK2 Derivative cells were infected with EV71 (H or 1095) (100 CCID_50_) and immediately treated with increasing concentrations of micafungin. Four days after treatment, LLC-MK2 Derivative cells were assayed for viability using MTT reagent. The viability of DMSO-treated cells was considered to be 100 %. (TIF 93 kb) Additional file 5: Figure S5. Antiviral effect of micafungin on EV71 infection in LLC-MK2 Derivative cells. LLC-MK2 Derivative cells were infected with EV71 (1 MOI) and simultaneously treated with increasing concentrations of micafungin. Twenty hours post-infection, dsRNAs were stained by using specific antibody and visualized by FITC-conjugated secondary antibody. Nuclear DNA was also visualized by DAPI staining. Cells with the fluorescent signal of dsRNAs were counted, and their ratio relative to the total cells at each concentration was calculated for plotting. The number of infected DMSO-treated cells was considered to be 100 %. (TIF 83 kb) Additional file 6: Figure S6. Antiviral effect of micafungin on CVB3 infection in HeLa cells. (A) HeLa cells were infected with CVB3 (100 CCID_50_) and simultaneously treated with increasing concentrations of micafungin. Forty-eight hours after treatment, antiviral activity was determined by the reduction of the cytopathic effect in an MTT assay. Cell viability of DMSO-treated cells was set to 0 % and that of uninfected cells was set to 100 %. (B) Same cells treated with indicated concentrations of micafungin without CVB3 infection were also analyzed for cell viability by using MTT assay. (TIF 95 kb) Additional file 7: Figure S7. Antiviral effect of micafungin on three strains of human rhinoviruses. H1HeLa cells were infected with human rhinovirus type 14 (A), 21 (B), or 71 (C) (100 CCID_50_) and immediately treated with indicated concentrations of micafungin. Three days after compound treatment antiviral activity was determined by the reduction of cytopathic effect using MTT assay. Cell viability of DMSO-treated cells was set to 0 % and that of uninfected cells was set to 100 %. (TIF 100 kb)
