# Supplementary figures and images for: Antiviral activity of micafungin against enterovirus 71
Source: Virol J. 2016 Jun 13;13:99. doi: 10.1186/s12985-016-0557-8 (PMC4907259; doi:10.1186/s12985-016-0557-8)

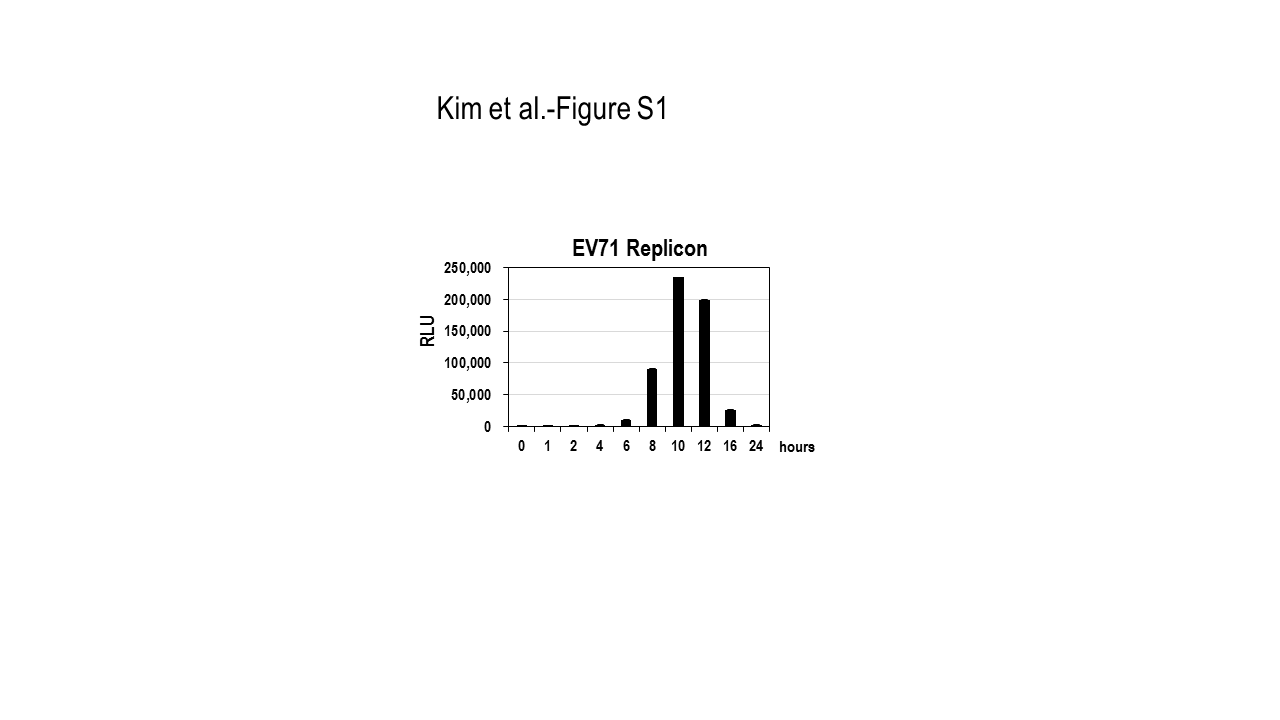

Supplement: Additional file 1: Figure S1. — Time-dependent luciferase activity of EV71 replicon in Vero cells. Vero cells were transfected with in vitro transcribed EV71-replicon RNAs and then firefly luciferase activity was measured at an interval of two hours. (TIF 80 kb) [file 12985_2016_557_MOESM1_ESM.tif]

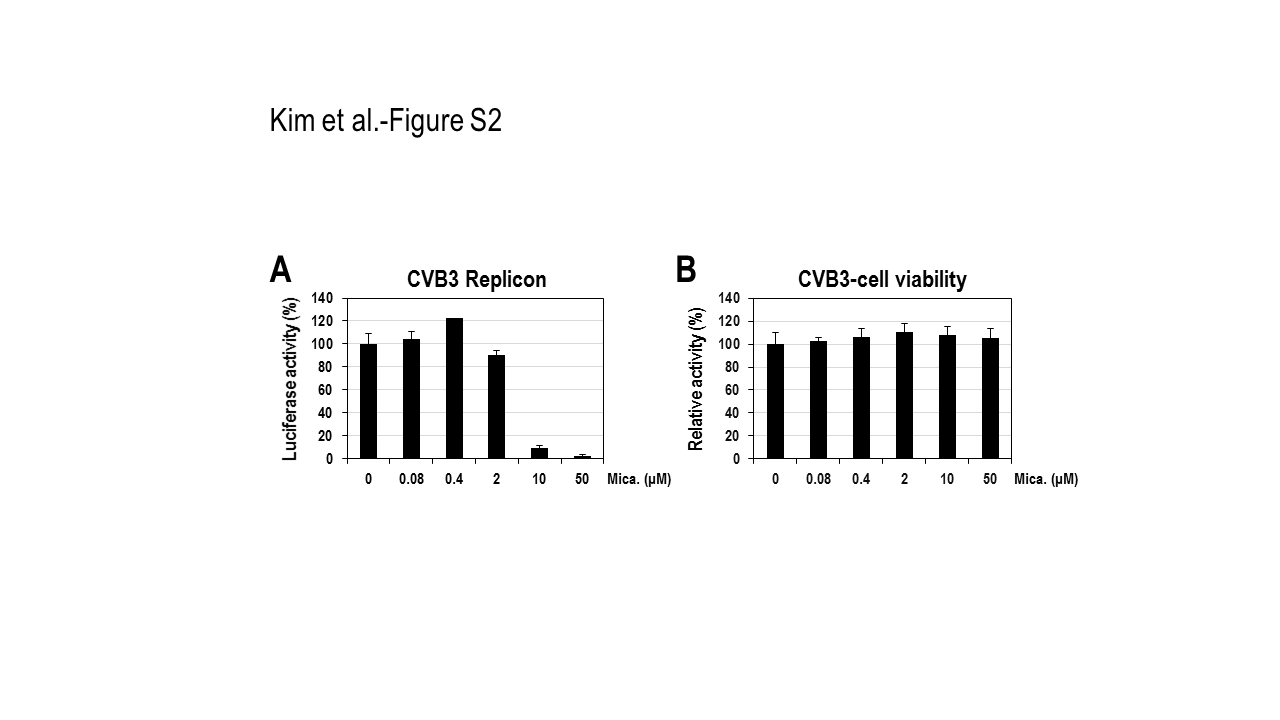

Supplement: Additional file 2: Figure S2. — Micafungin inhibits the replication of CVB replicon. (A) Vero cells were transfected with in vitro transcribed CVB3 replicon RNAs, instantly treated with indicated concentrations of micafungin for 8 hours and then assayed for firefly luciferase activity. Luciferase activity of DMSO-treated cells was set to 100 %. (B) At the same condition another set of CVB3 replicon-transfected cells were assayed for cell viability by using CellTiter-Glo reagent. Activity of DMSO-treated cells was set to 100 %. (TIF 100 kb) [file 12985_2016_557_MOESM2_ESM.tif]

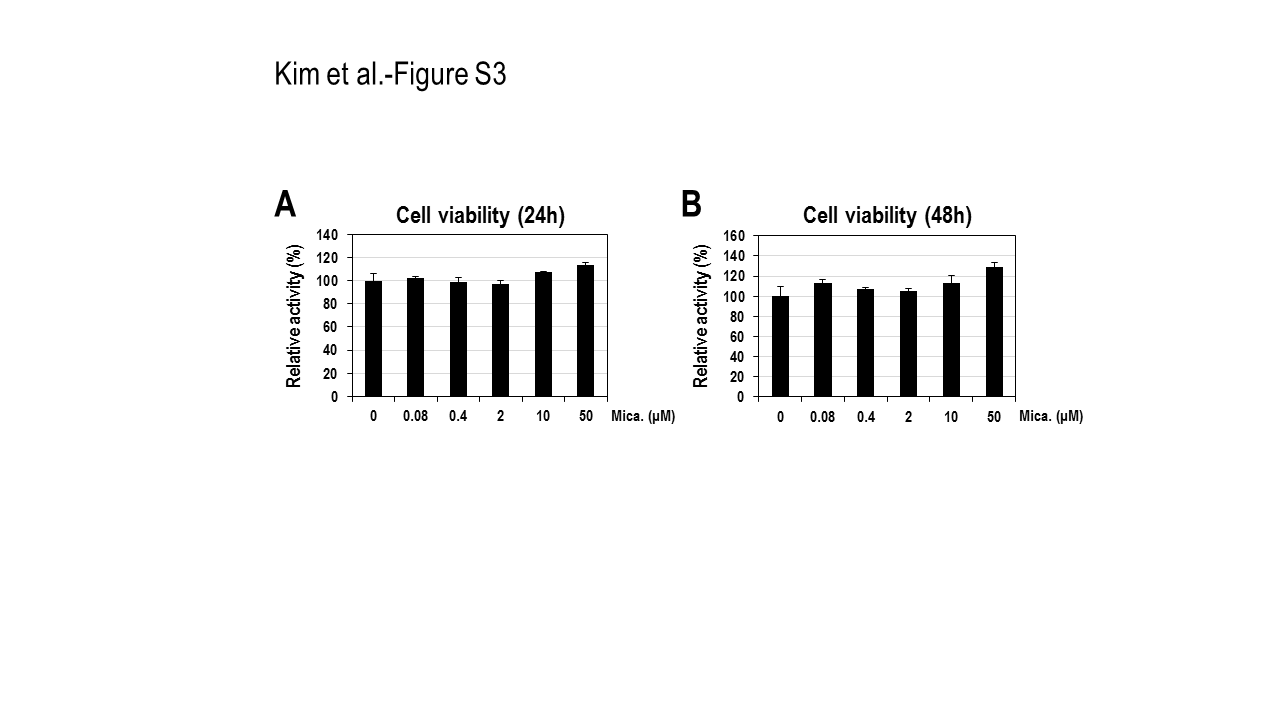

Supplement: Additional file 3: Figure S3. — Analysis of the cell toxicity of micafungin. Vero cells were treated with the indicated concentrations of micafungin for 24 h or 48 h and then assayed for viability using CellTiter-Glo reagent. The activity of DMSO-treated cells was considered to be 100 %. (TIF 98 kb) [file 12985_2016_557_MOESM3_ESM.tif]

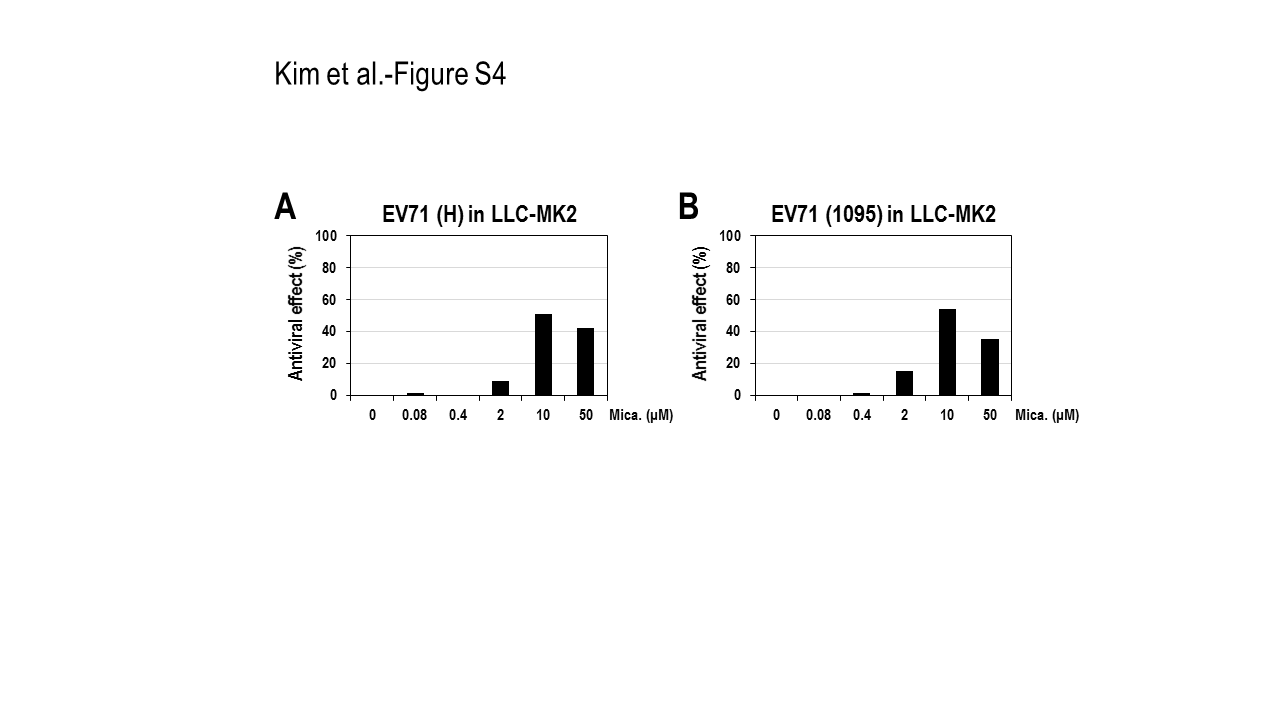

Supplement: Additional file 4: Figure S4. — Antiviral effect of micafingin on EV71 (strains H and 1095). LLC-MK2 Derivative cells were infected with EV71 (H or 1095) (100 CCID50) and immediately treated with increasing concentrations of micafungin. Four days after treatment, LLC-MK2 Derivative cells were assayed for viability using MTT reagent. The viability of DMSO-treated cells was considered to be 100 %. (TIF 93 kb) [file 12985_2016_557_MOESM4_ESM.tif]

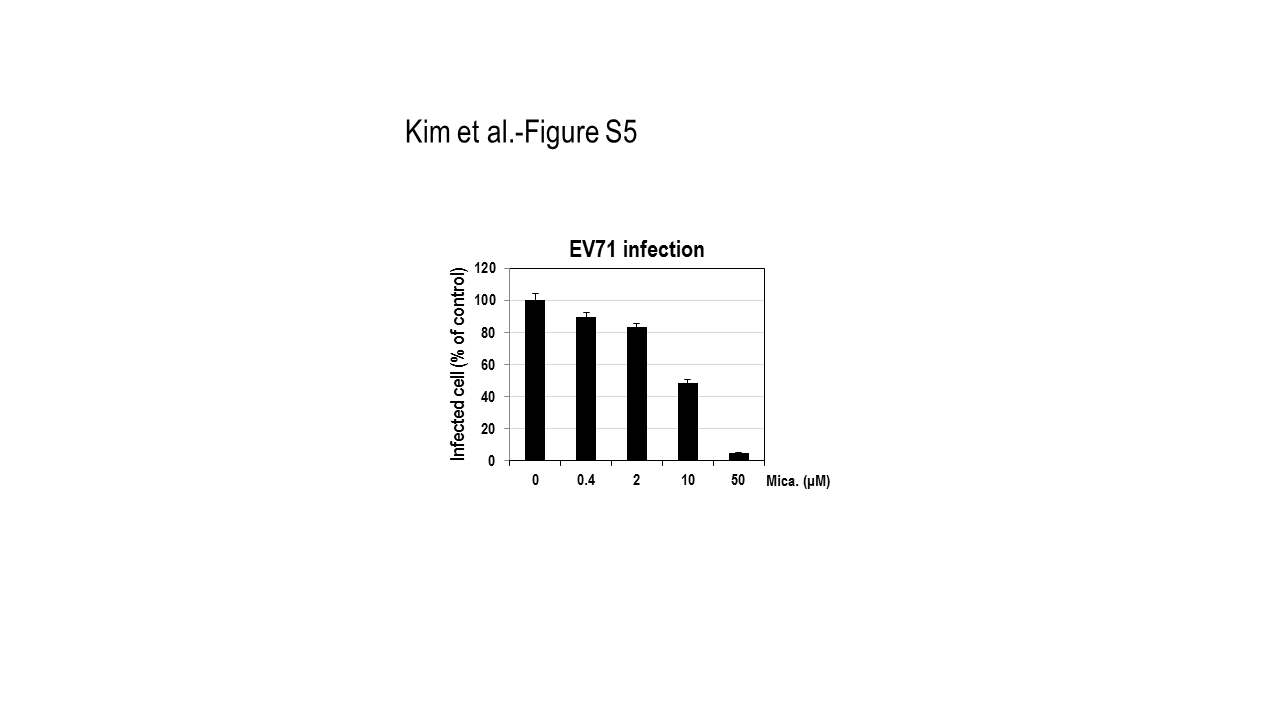

Supplement: Additional file 5: Figure S5. — Antiviral effect of micafungin on EV71 infection in LLC-MK2 Derivative cells. LLC-MK2 Derivative cells were infected with EV71 (1 MOI) and simultaneously treated with increasing concentrations of micafungin. Twenty hours post-infection, dsRNAs were stained by using specific antibody and visualized by FITC-conjugated secondary antibody. Nuclear DNA was also visualized by DAPI staining. Cells with the fluorescent signal of dsRNAs were counted, and their ratio relative to the total cells at each concentration was calculated for plotting. The number of infected DMSO-treated cells was considered to be 100 %. (TIF 83 kb) [file 12985_2016_557_MOESM5_ESM.tif]

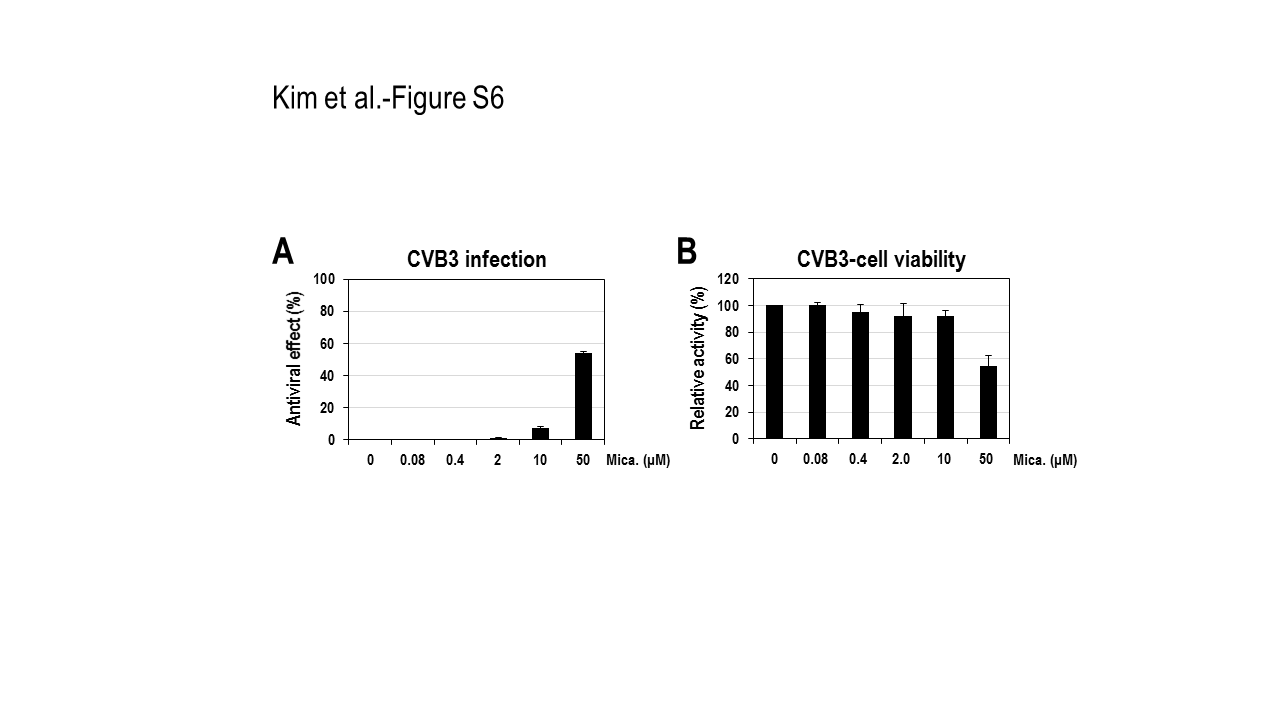

Supplement: Additional file 6: Figure S6. — Antiviral effect of micafungin on CVB3 infection in HeLa cells. (A) HeLa cells were infected with CVB3 (100 CCID50) and simultaneously treated with increasing concentrations of micafungin. Forty-eight hours after treatment, antiviral activity was determined by the reduction of the cytopathic effect in an MTT assay. Cell viability of DMSO-treated cells was set to 0 % and that of uninfected cells was set to 100 %. (B) Same cells treated with indicated concentrations of micafungin without CVB3 infection were also analyzed for cell viability by using MTT assay. (TIF 95 kb) [file 12985_2016_557_MOESM6_ESM.tif]

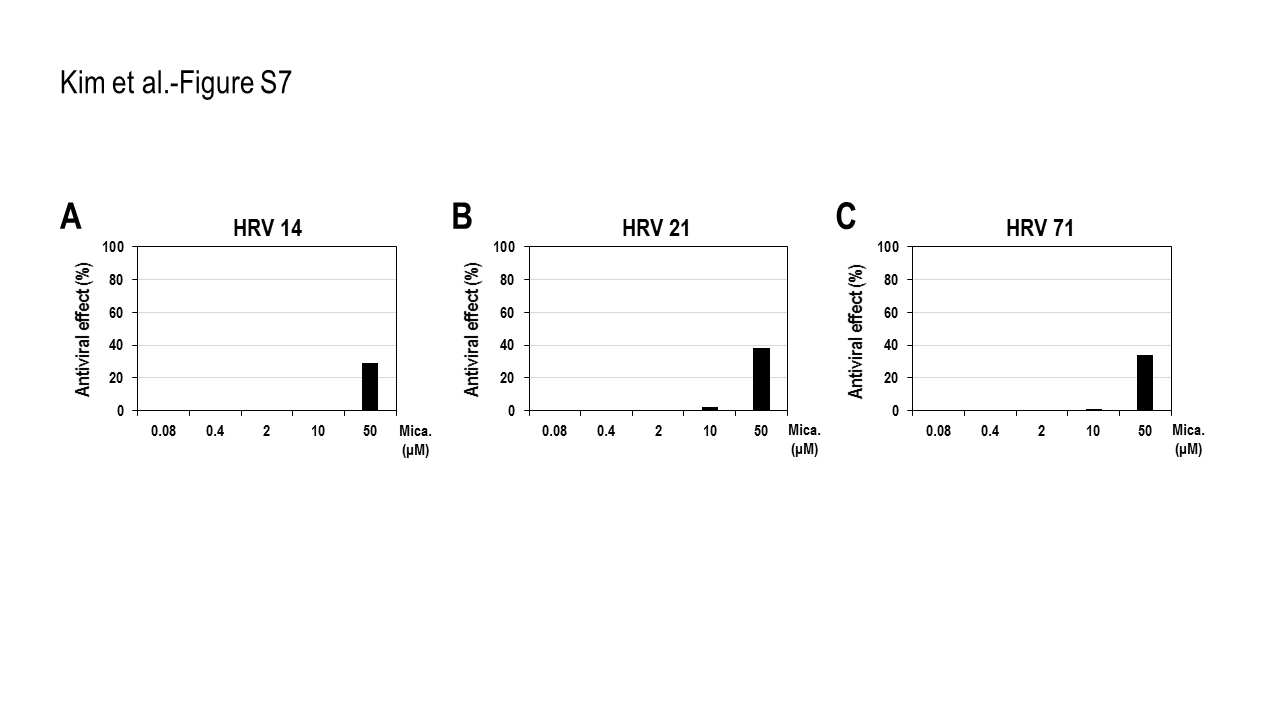

Supplement: Additional file 7: Figure S7. — Antiviral effect of micafungin on three strains of human rhinoviruses. H1HeLa cells were infected with human rhinovirus type 14 (A), 21 (B), or 71 (C) (100 CCID50) and immediately treated with indicated concentrations of micafungin. Three days after compound treatment antiviral activity was determined by the reduction of cytopathic effect using MTT assay. Cell viability of DMSO-treated cells was set to 0 % and that of uninfected cells was set to 100 %. (TIF 100 kb) [file 12985_2016_557_MOESM7_ESM.tif]
